# Management of Agitation in Huntington’s Disease: A Review of the Literature

**DOI:** 10.7759/cureus.9748

**Published:** 2020-08-14

**Authors:** Garrett Rossi, Joan C Oh

**Affiliations:** 1 Psychiatry, Cooper University Hospital, Camden, USA; 2 Psychiatry and Behavioral Sciences, Cooper Medical School of Rowan University, Camden, USA

**Keywords:** agitation, huntington disease, neurodegenerative disease, neuropsychiatric symptoms

## Abstract

Huntington’s disease (HD) is a rare neurodegenerative disease of the central nervous system characterized by choreatic movements, behavioral disturbances, and neuropsychiatric sequelae. The disease is inherited in an autosomal dominant fashion by an increased number of CAG repeats on the short arm of chromosome 4p16.3 in the Huntingtin gene. Huntington’s disease demonstrates the genetic principle of anticipation, where the larger the number of CAG repeats the earlier the signs and symptoms of the disease appear in subsequent generations. The symptoms often consist of behavioral disturbances and learning disturbances. The disease is suspected based on signs and symptoms and confirmed by genetic testing. There is no cure for the disease, and there is a high rate of neuropsychiatric symptoms including depression, and aggressive behavior. A significant risk of suicide in this population exists given the severity and unrelenting nature of the disease. Most patients will have multiple hospitalizations during the course of the illness. A consultant psychiatrist may be asked to evaluate and make recommendations for the treatment of acute agitation in HD patients. This can be a challenging task given the limited number of studies and the complex nature of agitation in the hospital setting. The aim of this review is to look at the currently available data for the treatment of acute agitation in patients with Huntington’s disease.

## Introduction and background

Changes in behavior are a common feature of Huntington’s disease (HD) and can cause distress to the patient as well as caretakers. Most patients with HD will experience multiple neuropsychiatric symptoms during the course of the illness [1-3]. HD is a rare genetic disorder, and thus, there is limited data available to provide evidence-based recommendations to treat the neuropsychiatric sequelae of the disease. 

One common neuropsychiatric symptom is agitation. Agitation is a common reason for psychiatric consultation on the medical floor. Agitation is an array of symptoms and behaviors that are commonly seen in patients with psychiatric or neurological disorders. It can rapidly escalate and can be categorized as mild, moderate, and severe. Episodes of acute agitation are considered psychiatric emergencies and often require pharmacological and physical intervention. There are a number of well-established behavioral and pharmacological interventions that can be used to intervene on most agitated patients on the medical floor. However, when patients are suffering from severe neurological diseases such as HD, they may not respond as well to these treatments and may have built up a tolerance from chronic administration of medications used to treat agitation.

Prior studies demonstrate a prevalence of agitation of 22-66% [1]. It’s important to note that in the studies available for psychotropic medications used to treat agitation, sample sizes are small [4]. Given the lack of standardized guidelines, treatment approaches should be individualized for agitated patients. The predominant clinical features of HD, stage of illness, and accompanying neuropsychiatric features should be taken into account when deciding on which treatment to use [4]. Although there is no cure for HD, it is not unreasonable to attempt to manage the neuropsychiatric symptoms commonly encountered in clinical practice. This review will look at current evidence-based treatment options of acute agitation in patients with established HD. 

## Review

Overview of agitation in Huntington’s disease

Agitation can occur at any time during the course of the disease. Agitation is defined as inappropriate behavior in a particular context characterized by excessive motor or verbal activity that may include physically aggressive behavior, restlessness, or pacing [1]. HD itself can be a source of agitation, but other potential sources of agitation, such as pain, should be considered and assessed clinically. Most patients with later-stage HD have impairments with regard to performing motor tasks, cognitive tasks, and activities of daily living such as feeding and bathing. Their inability to communicate effectively may mean that basic needs such as hunger and thirst are not met adequately, which can lead to agitation. Agitation can also be mistaken for akathisia, which is a subjective feeling of inner restlessness often seen as a side effect of medications used to treat agitation secondary to dopamine blockade. The best initial step in treating potential agitation in HD is to rule out any coexisting medical conditions, or pain that could be causing agitated behavior. Depending on the severity of the agitation, several pharmacological, behavioral, and procedural methods can be used to control agitation in the clinical setting. Clinicians should keep in mind that many of the side effects of these medications can mimic symptoms of HD and further complicate the clinical picture. 

General principles

❖ Identify and adequately treat any comorbid medical conditions that are known to precipitate agitation. 

❖ Potential medical conditions that can result in agitation include inadequate pain management, infectious diseases, metabolic derangements, drug interactions or drug side effects, comorbid substance use disorders, and comorbid psychiatric disorders. 

❖ Early identification of acute exacerbation of psychiatric symptoms (irritability, or any other coexisting psychiatric symptoms) or sleep disturbance in HD can prevent agitation and should be promptly identified and treated. 

❖ Modification of environmental stimulus such as noise reduction, limiting the number of disturbances by staff for things such as blood draws, placing a sitter or 1:1 companion in the room, making sure basic comfort needs are met such as positioning in the bed, access to food and water, and adequate location for voiding. 

Behavioral modifications

❖ Ensure that staff and 1:1 companion are aware of behavioral strategies to reduce or prevent agitation.

❖ If the patient becomes agitated, the initial response should be to reduce environmental stimulus, provide a safe, quiet space, allow the patient time to calm down with verbal de-escalation before intervening with pharmacotherapy, and provide gentle redirection and support.

Pharmacological interventions for agitation in HD

At this time, the preferred method for the treatment of agitation in HD is the use of benzodiazepines and various antipsychotic medications. Other methods have also been explored and include the use of mood-stabilizing medications in patients with chronic distress or threats of harm to self and others. Antipsychotic medications are a mainstay in the treatment of psychosis and agitation in HD, especially when psychosis, depression, or aggressive behavior is present. Both atypical and typical antipsychotics are being used, with atypical drugs being used more often. Atypical antipsychotics have fewer motor side effects than typical agents; thus, they are currently the preferred choice. Atypical antipsychotics have lower affinity for dopamine receptors and antagonize serotonin receptors leading to reduced side effect burden. However, the use of these agents demonstrated variable outcomes in managing aggression and agitation [4].

Olanzapine is one of the most commonly prescribed dopamine antagonists for the treatment of behavioral symptoms in HD [5]. Olanzapine is an atypical antipsychotic with a high affinity for serotonergic receptors (5HT2A, 5HT2c, 5HT3) as well as D2 antagonism [6]. Fluvoxamine or estrogens (CYP1A2 inhibitors) may increase plasma concentrations. Carbamazepine, omeprazole, and rifampin (CYP3A4 inducers) may reduce plasma concentrations [6]. In a six-month open-label trial, 11 patients with HD received 5mg of olanzapine with significant improvement in behavioral sub-scores for depression, anxiety, and irritability [7]. Reported dosages of olanzapine as a treatment for agitation vary between 5mg and 10mg daily. The main side effects of olanzapine include somnolence, orthostatic hypotension, hyperglycemia, dry mouth, weight gain, constipation, dyspepsia, increased appetite, prolonged QT interval, and tremor [6]. Olanzapine has the added benefit of reducing choreiform movements. The dopamine D2 antagonist properties of olanzapine may explain its possible benefits in the improvement of chorea [8]. Another study evaluated the effects of olanzapine in nine HD patients with primary psychiatric symptoms. The mean olanzapine dose was 11.4 mg/day, and the mean duration was 9.8 months. A reduction of 6.5 units was noted on the unified Huntington’s disease rating scale (UHDRS) but was not statistically significant [8,9]. 

Risperidone has evidence to support its use for treating psychiatric and possibly motor symptoms associated with HD [10]. Dosages of 2mg/day-4mg/day appear safe and effective for the treatment of agitation and psychosis in patients with HD [11]. A retrospective review of a group of HD patients treated with risperidone (average dose was 2.5mg) for an average of 15 months showed an improvement in psychiatric functioning, measured with the UHDRS behavioral score [9,11]. Concurrent use of risperidone may produce extrapyramidal symptoms. The most frequent adverse effects observed with risperidone include insomnia, anxiety, agitation, headache, weight gain, and nausea and vomiting [12].

Aripiprazole is an atypical antipsychotic medication that acts as a partial agonist at the dopamine receptors. One case report detailed the use of 10 mg/day titrated to 20 mg/day of aripiprazole for the treatment of psychosis and chorea in an HD patient [13]. There was a significant improvement in two weeks with respect to choreiform movements and psychosis with the above doses [13]. Another advanced symptomatic patient with agitation showed an improvement of agitation with aripiprazole (15 mg/day), with good control of motor symptoms without adverse effects [14]. Aripiprazole is a dopamine neurotransmitter stabilizer, acting as a functional antagonist in hyperdopaminergic states, and conversely, as a functional agonist in hypodopaminergic states. This may explain how aripiprazole controlled the involuntary movements related to HD by antagonizing dopamine receptors. One case report mentioned that switching to aripiprazole resolved a possible olanzapine-induced metabolic dysfunction in one patient [14]. This may be a better choice of medication for patients at high risk for metabolic dysfunction.

Quetiapine, an atypical antipsychotic medication known to have minimal extrapyramidal side effects, is a seemingly good choice for the treatment of behavioral disturbances in HD patients. Quetiapine is known to have a low affinity for the D2 receptor, theoretically minimizing some of the potential side effects. There was a noted improvement in behavioral symptoms (psychotic symptoms, agitation, irritability, and insomnia) without a worsening of motor function in one case series [15]. Quetiapine has also been used off-label to treat agitation, aggression, and psychosis in patients with dementia, ICU patients with agitation, and psychosis in Parkinson’s disease [16]. 

A case report detailed the use of intramuscular zuclopenthixol and medroxyprogesterone acetate in the treatment of agitation for patients with HD [17]. The case demonstrated sustained improvement in agitation and aggression. This article highlighted the potential for the use of other long-acting injectable antipsychotic medications as a possible intervention for HD patients who cannot take oral medications or have compliance issues. Another case report showed that intramuscular (IM) zuclopenthixol was more effective than paliperidone in the management of aggression [18]. It is suggested that zuclopenthixol has greater dopaminergic blockade in the mesocortical pathway, making it effective with less motor adverse effects [18].

Selective serotonin reuptake inhibitors (SSRIs) are a common treatment for depression and anxiety in patients with HD. Several studies have looked at the role of Fluoxetine, a selective serotonin reuptake inhibitor (SSRI), for treating aggression and agitation in HD. There was no change in baseline total functional capacity, neurological, or cognitive ratings between placebo and those treated with 20 mg/day of fluoxetine [19]. Although there was a slight reduction in agitation, it did not contribute to improved function. Fluoxetine failed to provide meaningful benefits in non-depressed HD patients [19]. Previous studies have indicated that the pathogenesis of Huntington’s disease may be mediated in part by the loss of brain-derived neurotrophic factor (BDNF) [20]. Antidepressants selectively block serotonin reuptake, which can increase BDNF levels and neurogenesis. One study reported that sertraline, an antidepressant, prolongs survival, improves motor performance, and ameliorates brain atrophy in the R6/2 HD mouse model [20]. Their findings provided evidence that sertraline may be neuroprotective in this HD model. There have been successful reports of treating human HD patients with sertraline [20]. 

There is a recent interest in the use of medical cannabis for movement and neurodegenerative disorders [21]. Three trials looked at the efficacy of cannabinoids in HD with a total of 84 participants in these trials [22-24]. With respect to these three trials, no significant improvement in the movement disorder itself or the neuropsychiatric sequelae over placebo were established. 

Neuromodulation interventions for agitation in HD

Neuromodulation techniques such as electroconvulsive therapy (ECT) can be considered for refractory cases of agitation. Electroconvulsive therapy (ECT) has been long used as a treatment for severe or medication-resistant mood disorders and catatonia. There is limited data on the use of ECT to treat agitation in HD. However, ECT has been used successfully to treat agitation in other psychiatric conditions, including major neurocognitive disorder. The literature is primarily limited to case reports and case series at this time. 

A recent case report detailed the use of ECT to treat agitation in patients with HD, whose agitation was refractory to pharmacotherapy [25]. They noted that after three sessions, the patient’s behavior was improved with significantly decreased verbal and physical aggression [25]. By the 5th session, the patient’s agitation was at baseline, and treatment was stopped at that point. The patient remained in her usual state of health for two months after ECT treatment, demonstrating the lasting benefits of this neuromodulation technique. ECT has been shown to treat agitation in other conditions, such as treatment-resistant schizophrenia and major neurocognitive disorder. There are a few but encouraging number of reports on the efficacy of ECT for psychiatric disorders with HD. ECT enhances dopaminergic, serotonergic, adrenergic, glutaminergic, and GABAergic neurotransmission. In HD, ECT has been used to target mood symptoms and psychosis. Treating agitation in HD with ECT has not been well studied. It is recommended to consider using ECT after a failed trial of antipsychotics with mood stabilizers, and the use of maintenance ECT for one month [26]. A recent case report in 2017 described an HD patient who developed worsening movement disorder symptoms after undergoing ECT for depression [27]. Clinicians should be mindful of the possibility of this side effect.

## Conclusions

The body of literature regarding agitation in HD is limited in both quality and quantity of available research. The above information is intended to help guide clinical decisions when managing acute agitation in patients with HD. The data provides behavioral interventions, pharmacological interventions, and neuromodulation techniques that have been shown to help patients with HD and agitation. As with any recommendations clinical judgement is of the utmost importance. Some common things that should be done in all cases include obtaining information from the primary team, patient and if possible, a caretaker. Often people with HD have impaired awareness due to their condition especially if agitation is present. It’s important to identify and treat any co-morbid medical conditions, psychiatric co-morbid conditions, and identify potential environmental triggers. Identifying and treating any psychiatric conditions will reduce the potential for more severe neuropsychiatric disturbances. This may even provide a preventative strategy for patients at risk for agitation. The goal in the treatment of agitation should be safety of the patient/staff, and this includes limiting polypharmacy. It is best to select medications with the best side effect profile for the case, considering the possibility for worsening cognitive and motor symptoms of HD with many medications used to treat agitation. Psychoeducation should be provided to caregivers about the potential causes and treatment of agitation. Forming a good therapeutic alliance and improving understanding of signs and symptoms will help identify agitation earlier and possibly prevent it. 
